# Evaluation of Diagnostic Accuracy of Eight Commercial Assays for the Detection of Rubella Virus-Specific IgM Antibodies

**DOI:** 10.1128/JCM.01597-21

**Published:** 2022-01-19

**Authors:** Joanne Hiebert, Vanessa Zubach, Carmen L. Charlton, Jayne Fenton, Graham A. Tipples, Kevin Fonseca, Alberto Severini

**Affiliations:** a Viral Exanthemata and STD Section, National Microbiology Laboratory, Public Health Agency of Canada, Winnipeg, Manitoba, Canada; b Public Health Laboratory, Alberta Precision Laboratories, Edmonton, Alberta, Canada; c Department of Laboratory Medicine and Pathology, University of Alberta, Edmonton, Alberta, Canada; d Li Ka Shing Institute of Virology, University of Alberta, Edmonton, Alberta, Canada; e Department of Medical Microbiology and Immunology, University of Alberta, Edmonton, Alberta, Canada; f Department of Microbiology, Immunology and Infectious Disease, University of Calgary, Calgary, Alberta, Canada; g Department of Medical Microbiology and Infectious Diseases, Faculty of Health Sciences, University of Manitoba, Winnipeg, Manitoba, Canada; Cepheid

**Keywords:** rubella IgM serology, ELISA, EIA, CLIA, sensitivity, specificity, clinical accuracy, IgM, kit performance, rubella, serology

## Abstract

Rubella and congenital rubella syndrome are caused by the rubella virus and are preventable through vaccination, making disease eradication possible. Monitoring of progress toward global eradication and local elimination requires high-quality, sensitive disease surveillance that includes laboratory confirmation of cases. Previous evaluations of anti-rubella IgM detection methods resulted in the broad adoption of the Enzygnost (most recently manufactured by Siemens) enzyme-linked immunosorbent assay (ELISA) kits within WHO’s global measles and rubella laboratory network, but they have been discontinued. This study evaluated seven comparable ELISAs from six manufacturers (Trinity Biotech, Euroimmun, Clin-Tech, NovaTec and Virion\Serion) as well as one automated chemiluminescent assay (CLIA) from DiaSorin. These assays include three IgM capture assays and five indirect ELISAs. A panel of 238 sera was used for the evaluation that included 38 archival rubella IgM-positive sera and 200 sera collected from patients with symptomatically similar diseases, such as measles, dengue, parvovirus B19 infection, and roseola. With this panel of sera, the sensitivity of the methods ranged from 63.2% to 100% and the specificity from 80.0% to 99.5%. No single method had both sensitivity and specificity of >90%, unless sera with equivocal results were considered presumptively positive. Some assays, particularly the Serion ELISA, had a large number of false positives with parvovirus B19 IgM-positive sera as well as sera from confirmed measles cases. The performance characteristics identified in this evaluation serve as a reminder to not rely solely on rubella IgM results for case confirmation in elimination settings.

## INTRODUCTION

Rubella and congenital rubella syndrome are caused by the rubella virus and are preventable through vaccination. Disease eradication is possible through sustained high vaccination coverage, and elimination of endemically circulating virus has been achieved in many countries globally, including Canada (1, 2). Monitoring of progress toward global eradication and local elimination requires high-quality, sensitive disease surveillance that includes laboratory confirmation of cases (1, 3, 4). Historically, this was often achieved through detection of rubella-specific IgM antibodies, although this has been augmented or replaced by virus detection by reverse transcription-PCR (RT-PCR), particularly in countries approaching or having achieved elimination (3–6). Nonetheless, IgM serology remains an important tool in many countries where it can be used for case confirmation (settings of endemicity) and active disease surveillance (case finding in elimination settings) through reflex testing or cotesting of sera captured through other surveillance systems, such as those for measles and arboviruses (7, 8). It is critical that validated methods with high specificity and sensitivity be employed. Previous evaluations of anti-rubella virus IgM detection methods resulted in the broad adoption of Enzygnost (most recently manufactured by Siemens) enzyme-linked immunosorbent assay (ELISA) kits within WHO’s global measles and rubella laboratory network (8, 9). These kits have been discontinued; thus, there is a need to identify replacement methods (10).

This study evaluated seven comparable ELISA methods from six manufacturers (Trinity Biotech, Euroimmun, Clin-Tech, NovaTec, and Virion\Serion) as well as one automated chemiluminescent immunoassay (CLIA) from DiaSorin. A panel of 238 sera was assembled that included, in the non-rubella panel, sera that were IgM positive for chikungunya virus, dengue virus, measles virus, parvovirus B19, roseola virus, and zika virus. These viruses were chosen because they cause illnesses that can also present with fever and rash symptoms and thus may be captured in rubella testing algorithms during differential diagnosis or active case finding (7, 8, 11–15). Some, such as parvovirus B19, have been previously noted to be a source of cross-reactivity in methods for the detection of anti-rubella virus IgM antibody (16).

## MATERIALS AND METHODS

### Panel sample set.

The study was conducted retrospectively and employed anonymized residual sera that had been received either at the National Microbiology Laboratory (NML) or the Alberta Public Health Laboratory (ProvLab) for serological testing (convenience sampling). In addition, 36 sera, available as part of the inventory of the NML’s rubella serology proficiency panel program, were included (Table 1). These were archival sera sourced from a variety of suppliers, including commercial rubella IgM-positive controls. To control for their storage prior to this study (duration, conditions, and number of freeze-thaws), the majority result was used to define their expected result, and sera without a positive result in most methods were excluded from the analysis. As a result, one specimen was excluded.

**TABLE 1:** Characteristics of the serum samples included in this study

Sample group	Source	No. of patients	No. of serum samples (no. included)	Median age, yrs (range)
Rubella sera				
Rubella IgM positive	Leftover sera from proficiency panel program; includes commercial sera	Unknown	36 (35)^*a*^	Unknown
Probable post-MMR reactions	Sera submitted for suspected primary HHV-6	2	0 (2)^*b*^	1 (1–1)
	Sera from measles outbreaks	1	0 (1)^*b*^	1
Total rubella sera			36 (38)^*a*^^,^^*b*^	
Non-rubella sera				
Chikungunya IgM positive	Sera submitted for suspected chikungunya infection	4	4	41 (40–56)
Dengue IgM positive	Sera submitted for suspected dengue infection	Unknown	34	Unknown
Fever and rash of unknown etiology	Sera submitted for suspected primary HHV-6; HHV-6 IgM negative and IgG positive	37	37	7 (0–67)
Confirmed measles cases	Pooled sera from measles outbreaks	68	49 (48)^*b*^	15 (1–53)
	Commercial serum supplier; acute measles infection	Unknown	1	Unknown
Parvovirus B19 IgM positive	Sera submitted for suspected parvovirus B19	35	35	36 (7–50)
Confirmed roseola cases	Sera submitted for suspected primary HHV-6; IgM positive	24	24 (22)^*b*^	1 (0–3)
	Sera submitted for suspected primary HHV-6; IgM and PCR positive	16	16	0.5 (0–2)
Zika IgM positive	Sera submitted for suspected zika virus infection	3	3	30 (26–52)
Total non-rubella sera			203 (200)^*b*^	14 (0–67)
Total panel sera			239 (238)^*a*^^,^^*b*^	11 (0–67)

a Thirty-six archival sera with a history of rubella IgM positive results were tested, but to control for specimen quality, the majority result was used to define their expected result. As a result, one specimen was excluded from the analysis.

b Three sera from confirmed roseola and measles cases were determined to represent probable post-MMR reactions and thus were reassigned to the rubella sera group.

A total of 166 residual clinical sera that were confirmed to be IgM positive or equivocal for other fever- and rash-causing viruses were included in the panel (Table 1). Sera from 68 laboratory-confirmed cases that met the national case definition for measles by local public health authorities and collected during measles outbreaks were included (17). However, the residual volume of all specimens was insufficient for use in the Liaison method, which requires at least 170 μL. As a result, the sera were pooled to create a total of 49 specimens with volumes of 170 to 190 μL. An additional specimen, sourced from a company that supplies sera to proficiency panel providers, with known acute measles infection status was included in the confirmed measles panel (sample number 357006; Gesellschaft für Biotechnologische Diagnostik mbH, Berlin, Germany).

Additional agents included were chikungunya virus (*n* = 4), dengue virus (*n* = 34, 3 of which were pooled), human herpesvirus 6 (HHV-6) (*n* = 40, 16 of which were also PCR positive), parvovirus B19 (*n* = 35), and zika virus (*n* = 3).

An additional 37 sera from clinical cases with fever and rash, as recorded on the test requisition, and referred for HHV-6 serology with HHV-6 IgM-negative IgG-positive results were included.

The sera for which collection dates were known were collected between 2001 and 2015.

The final panel (*n* = 239) was assembled, randomized, and blinded at the NML. The panel was frozen and sent to the Alberta provincial laboratory for testing on the automated Liaison platform. Upon completion, the panel was refrozen and returned to the NML for all manual plate-based ELISAs.

### Liaison chemiluminescent assay.

The Liaison rubella IgM assay is a high-volume commercial platform which uses inactivated viral particles in a chemiluminescent immunoassay. A single technologist at the Public Health Laboratory in Alberta ran all samples according to the manufacturer’s instructions. Calibrators and instrument controls were within range for all specimens tested. An external positive control was included in all assays in triplicate. Due to volume requirements and limiting volume available for some panel specimens, 175 of the 239 panel specimens had sufficient volume for testing on the Liaison platform.

### ELISA serological methods.

The following commercial ELISA kits for the detection of rubella IgM were included in the evaluation: Captia, Enzygnost, Euroimmun (native antigens and recombinant glycoprotein), Microimmune, NovaLisa, and Serion (details are provided in Table 2). All methods were performed by a single technician at the NML and according to the manufacturer’s instructions for use (IFU) provided in the kits, except for the volume of serum used if it exceeded 5 μL in the IFU, due to the limited volume available. For those kits (Enzygnost, Euroimmun, Euroimmun glycoprotein, NovaLisa, and Serion), the volume of serum and the serum dilution buffer (to maintain the dilution ratio given in the IFU) used was reduced by one half. The same external positive control was included in all ELISA methods and all test plates, in duplicate, or singly for the Captia kit. Washing steps were automated on a BioTek 50TS 96-well plate washer. Temperatures (room temperature and 37°C incubator) were verified with calibrated thermometers to be within the limits given in the kit IFU prior to performing the tests. Optical densities were read as per the IFU with a Tecan Sunrise microplate absorbance reader. Optical density data were exported to a Microsoft Excel 2016 file and then copied into custom-made, verified Microsoft Excel 2016 templates, where calculations and result determinations were automated. Test plate validation and specimen results were determined as per the manufacturers’ IFU. The Serion kit IFU included three possible methods of generating a qualitative result, and all methods were followed. Samples with equivocal results were repeated for Captia and Microimmune, as advised in the IFU. All ELISA kits were tested in a single freeze-thaw cycle of the panel, with the exception of the repeats of equivocal results and the Captia kit.

**TABLE 2:** Characteristics of the commercial kits for the detection of anti-rubella IgM antibodies evaluated in this study^*a*^

Characteristic	Captia (Trinity Biotech USA, Jamestown, NY)	Enzygnost (Siemens Healthcare Diagnostics Products GmbH, Marburg, Germany)	Euroimmun (Euroimmun Medizinische Labordianostika AG, Lübeck, Germany)	Euroimmun Glycoprotein (Euroimmun Medizinische Labordianostika AG, Lübeck, Germany)	Liaison (DiaSorin Saluggia [VC], Italy)	Microimmune (Clin-Tech Limited, Guildford, UK)	NovaLisa (NovaTec Immundiagnostica GmbH, Dietzenbach, Germany)	Serion Classic (Institut Virion\Serion GmbH, Würzburg, Germany)
Catalogue no.	2325360	10446585	EI 2590-9601 M	EI 2590-9601-2 M	310730	RuVM014	RUBM0400	ESR129M
Lot no. evaluated	095	48481 and 48628	E190508AS	E190625BM	178019	K91-4-2 and K91-192-1	RUBM-148	SBK.DE
Version no. of IFU evaluated	5360-29 Rev L 03/2015	2017-04	06/01/2017	12/06/2019	CAEN-200/007-047, 04-2016-09	K50p201801	20082019-RUO-Ka	V 129.17
European regulatory status	CE IVD	CE IVD	CE IVD	CE IVD	CE IVD	RUO	RUO	CE IVD
Health Canada clearance	Yes	Yes	Yes	Yes	Yes	No	No	No
US FDA clearance	Yes	No	No	No	Yes	No	No	No
Method description	Indirect ELISA	Indirect ELISA	Indirect ELISA	Indirect ELISA	Automated IgM capture CLIA	IgM capture (anti-IgM antibody coated wells)	IgM capture (anti-IgM antibody coated wells)	Indirect ELISA
Antigen	Purified rubella antigen	Paired whole virus and control (cellular) antigen wells	Whole virus	Recombinant rubella glycoprotein	Inactivated rubella viral particle	Recombinant rubella	Whole virus	Not specified
Use of RF absorbent	Yes but no additional incubation	Yes, separate incubation step	Yes, separate incubation step	Yes, separate incubation step	No	No	No	Yes, separate incubation step
Incubation conditions	Room temp (21–25°C)	37°C, humidified	Room temp (18–25°C)	Room temp (18–25°C)	NA; completely automated	37°C, humidified	37°C	37°C, humidified
No. of reagents to prepare	1	4	1	1	0	2	1	2
No. of incubation steps	3	4	4	4	3	4	3	4
Total incubation time	1 h 15 min	2 h 45 min	1 h 25 min	1 h 25 min	NA; completely automated	3 h 20 min	1 h 45 min	2 h 15 min
Approximate total time	1 h 30 min	3 h	1 h 40 min	1 h 40 min	Time to first result: 35 min	3 h 40 min	2 h	2 h 30 min
Correction for interrun and interlot variability	Yes, ratio of sample OD to in-run calibrator OD used; lot specific control ranges	Yes, correction factor based on in-run control and lot specific value applied to specimen ODs	Yes, ratio of sample OD to in-run calibrator OD used	Yes, ratio of sample OD to in-run calibrator OD used	No	No; fixed control ranges and cutoff values	Yes, ratio of sample OD to in-run cutoff control OD used	Yes, choice of 3 result determination methods and all use a correction method
Maximum no. of samples per plate (per kit)	91 (91)	45 (90)	93 (93)	93 (93)	100	44 (44)	92 (92)	92 (92)
Shortest reagent shelf life once opened	Same as kit expiry	2 mo (strips)	4 mo (strips)	4 mo (strips)	8 wks on board	3 mo (strips)	Same as kit expiry	6 mo
Completeness of kit	All reagents provided	Supplemental kit required (catalogue no. OUVP)	All reagents provided	All reagents provided	Controls separate (catalogue no. 310732)	All reagents provided	All reagents provided	RF absorbent separate (catalogue no. Z200)
Serum vol	10 μL, per the IFU; 5 μL was used	20 μL, per the IFU; 10 μL was used	10 μL, per the IFU; 5 μL was used	10 μL, per the IFU; 5 μL was used	20 μL used with minimum 150-μL dead vol	5 μL, per the IFU	10 μL, per the IFU; 5 μL was used	10 μL, per the IFU; 5 μL was used
Total cost per test sample for this study (USD)	$2.50	$5.61	$3.83	$4.50	$3.18	$9.30	$2.89	$5.05
No. of samples tested (no. of rubella samples)	238 (38)	238 (38)	238 (38)	238 (38)	174 (34)	238 (38)	238 (38)	238 (38)

a no., number; IFU, instructions for use provided in the kit; CE, Conformité Européenne mark (CE mark); IVD, *in vitro* diagnostic device; RUO, research use only; CLIA, chemiluminescent assay; ELISA, enzyme-linked immunosorbent assay; RF, rheumatoid factor; temp, temperature; NA, not applicable; mo, months; wks, weeks.

### Treatment of equivocal results.

All methods included an indeterminate or equivocal range where the result could not be categorized as either positive or negative. Results in these ranges were handled in two ways for assessment of test performance: an “always-wrong” approach and a “presumptively positive” approach. In both scenarios, equivocal results with the non-rubella sera were always considered positive. Thus, only one specificity value was calculated for each method. Sensitivity and accuracy were calculated using both approaches where the equivocal results for the rubella sera were considered negative (always wrong) or positive (presumptively positive). (For the accuracy calculations, equivocal results with the non-rubella sera were always considered positive.)

### Data analysis.

Microsoft Excel 2016 was used to compile results and to calculate sensitivity, specificity, and accuracy values and their 95% confidence intervals (CI). Confidence intervals were calculated using the score method, as follows: L (lower limit) = {2*np* + *z*2 − 1 − *z* √ [*z*2 − 2 − (1/*n*) + 4*p*(*nq* + 1)]}/2(*n* + *z*2) and U (upper limit) = {2*np* + *z*2 + 1 + *z* √ [*z*2 + 2 − (1/*n*) + 4*p*(*nq* − 1)]}/2(*n* + *z*2) where *z* = 1.96, *p* is sensitivity or specificity, and *q* = 1 − *p*. If *p* = 1, then U = 1, since specificity and sensitivity cannot be >100% (18).

An online calculator was used to calculate the kappa measure of agreement and 95% confidence intervals (GraphPad [https://www.graphpad.com/quickcalcs/kappa1/]).

GraphPad Prism 8.3.0 was used to generate receiver operating characteristic (ROC) curves and calculate the areas under the curves with confidence intervals.

Violin plots of the normalized output values for each of the 7 ELISA methods were generated in GraphPad Prism 8.3.0. Each ELISA method performed method-specific manipulations of the raw optical density (OD) values to generate a test output value (which was then used to determine the qualitative result). The scale of the resulting output values was not comparable across methods, particularly for the Serion activity calculator method, which generated values in the thousands, while most other methods had values of <10. Therefore, the data for each method were normalized to 100 to facilitate their comparison. In Microsoft Excel 2016, the grouped rubella and non-rubella output values were sorted from highest to lowest for each method. The highest value for each method was arbitrarily set to 100. To determine the normalized value, each value was divided by the highest value for the method and then multiplied by 100. The normalized values were used in the violin plots.

## RESULTS

### Panel samples.

A panel of 239 sera was assembled that included 36 archival sera from the NML’s rubella serology proficiency panel program inventory and 203 sera that were IgM positive for a number of other viruses (chikungunya virus, dengue virus, HHV-6 [roseola], measles virus, parvovirus B19, or zika virus) or were from patients who presented with fever and rash of unknown etiology (the “non-rubella” panel) (Table 1). To control for the history (age, storage conditions, etc.) of the archival rubella sera, the majority result was used to define inclusion in the evaluation; sera without a positive result in most methods were excluded from the analysis. As a result, one specimen was excluded.

This panel included a number of sera collected from individuals who were eligible to receive their first dose of rubella-containing vaccine, which in Canada is recommended at 12 months of age and is a combined measles, mumps, and rubella (MMR) vaccine or a measles, mumps, rubella, and varicella (MMRV) vaccine (19). Thus, it was possible that some of the sera included in the non-rubella panel were collected from recently vaccinated individuals. Rubella IgG avidity (Euroimmun, catalogue number EI 2590-9601-1G) was determined on all sera that had a positive or equivocal result on any of the evaluated methods. The results were reviewed to determine and control for the likelihood of recent vaccination in the non-rubella panel (data not shown). Two sera from the roseola subset and one from the measles subset, which were collected from infants 1 year of age, had low rubella IgG avidity. These three sera had rubella IgM-positive or -equivocal results in many of the tested methods and were also positive for anti-mumps virus IgM antibody (Euroimmun, catalogue number EI 2630–9601 M) (data not shown). As a result, all three sera were classified as probable post-MMR vaccine reactions.

In total, a panel of 238 sera were included in this evaluation, of which 38 sera were classified as rubella sera and 200 as non-rubella sera (Table 1). However, only 174 (34 rubella and 140 non-rubella sera) had sufficient volume to be included on the Liaison automated system.

### Method characteristics.

Several characteristics, such as choice of antigen, completeness of the kit components, length of time needed for the test, incubation temperatures and user-friendliness, of the methods that were included in the study were compared (Table 2). Excluding the automated Liaison method, most methods were indirect ELISAs, with two IgM capture methods: Microimmune and NovaLisa. The indirect ELISA kits all used rheumatoid factor (RF) absorbent. Where the antigen was specified, most ELISA kits used whole virus antigen while two used recombinant antigen. Notably, the Microimmune kit, like the Enzygnost kit, required the application of the test serum to a second control well. However, the Microimmune kit came with only one test plate and thus could test less than half the maximum number of specimens per kit that could be tested in other kits (including Enzygnost). The kits varied in user friendliness, as defined by number of reagents to prepare, number of incubation steps, number of test wells needed per specimen, and total test time, with the Enzygnost and Microimmune kits being less user friendly. While the Serion kit was more user friendly to perform, it had a more complex method of determining the test result, with the choice of three possible methods. One of these methods, the activity calculator, required the use of a complicated Excel template, obtained from Serion, that used 4-parameter logistic (4-PL) mathematical curve fitting to generate a quantitative value that was converted to a qualitative result with lot-specific cutoffs.

### Sensitivity.

The rubella serum panel, consisting of 38 (34 for Liaison) specimens (Table 1), was used to evaluate the sensitivity of the kits, calculated in two ways, with equivocal results considered negative or positive (Table 3). When equivocal results were considered negative, the calculated sensitivity ranged from 63.2% (NovaLisa) to 97.4% (Enzygnost and all three Serion result determination methods) which improved to 78.9% (NovaLisa) to 100% (Enzygnost and Serion) when equivocal results were considered positive. Only the Enzygnost and Serion methods had sensitivities above 90% when sera with equivocal results were considered negative. With a presumptively positive approach, the Euroimmun GP, Liaison, and Microimmune methods also had sensitivities over 90%. In either scenario, the Enzygnost and Serion methods were the most sensitive (97.4% or 100%) and were statistically significantly more sensitive than the NovaLisa method when sera with equivocal results were considered negative (Table 3).

**TABLE 3:** Results and calculated sensitivities, with equivocal results counted as either negative or positive, of the commercial methods for the detection of anti-rubella IgM antibodies evaluated with the rubella serum panel (*n* = 38)^*a*^

Assay	No. positive	No. equivocal	Sensitivity (%)^*b*^	95% CI (%)^*b*^	Sensitivity (%)^*c*^	95% CI (%)^*c*^
Captia	30	2	78.9	62.2–89.9	84.2	68.1–93.4
Enzygnost	37	1	97.4^*f*^	84.6–99.9	100.0	88.6–100.0
Euroimmun	28	6	73.7	56.6–86.0	89.5	74.3–96.6
Euroimmun glycoprotein	34	3	89.5	74.3–96.6	97.4	84.6–99.9
Liaison^*d*^	28	3	82.4	64.8–92.6	91.2	75.2–97.7
Microimmune	30	6	78.9	62.2–89.9	94.7	80.9–99.1
NovaLisa	24	6	63.2^*f*^	46.0–77.7	78.9	62.2–89.9
Serion (activity calculator)^*e*^	37	1	97.4^*f*^	84.6–99.9	100.0	88.6–100.0
Serion (OD range)^*e*^	37	1	97.4^*f*^	84.6–99.9	100.0	88.6–100.0
Serion (special case formula)^*e*^	37	1	97.4^*f*^	84.6–99.9	100.0	88.6–100.0

a CI, confidence interval.

b Specimens with equivocal results counted as negative.

c Specimens with equivocal results counted as positive.

d The number of rubella specimens tested with the Liaison method was 34, not 38.

e Three methods of sample result determination, using the single set of optical density data from the test plates, were provided in the manufacturer’s IFU. All three methods were evaluated.

f Significant difference (*P* < 0.05) from the most sensitive methods (all three Serion result determination methods and Enzygnost) based on nonoverlapping 95% confidence intervals. When equivocal results were counted as negative, the NovaLisa kit was significantly less sensitive than the Serion and Enzygnost kits. When equivocal results were counted as positive, there was no significant difference between the kits with respect to their calculated sensitivity.

### Specificity.

The results of testing with the non-rubella serum panel, which consisted of seven subsets of panels for a total of 200 specimens (Table 1), was used to calculate the specificity of the methods. Only unequivocally negative results were included in the specificity determination; equivocal results were included in the denominator (effectively counted as positive). The calculated specificity for the methods under evaluation ranged from 80.0% (Serion, OD range method) to 99.5% (NovaLisa) (Table 4). The reference method, Enzygnost, had a specificity of 89.5%, and six methods exceeded this level: Euroimmun (91.5%), Captia (95.0%), Euroimmun glycoprotein (96.0%), Liaison (97.9%), Microimmune (98.0%), and NovaLisa (99.5%). The method with the highest calculated specificity (NovaLisa, 99.5% with a 95% CI of 96.8 to 100%) was significantly better than Enzygnost (89.5% with a 95% CI of 84.2 to 93.2%), Euroimmun (91.5% with a 95% CI of 86.5 to 94.8%), and all three Serion result determination methods (specificity of 80.0% and 80.5% with 95% CI of 73.6 to 85.2% and 74.2 to 85.6%). A slight difference was noted between the three result determination methods provided with the Serion method: the OD range method resulted in one additional equivocal result, for a total of 40 positive or equivocal results for the panel of 200 nonrubella sera.

**TABLE 4:** Results and calculated specificities of the commercial methods for the detection of anti-rubella virus IgM antibodies evaluated with the non-rubella serum panel (*n* = 200), by subset^*a*^

Assay	No. of positive or equivocal results (% specificity)								95% CI of specificity
	Chikungunya virus (*n* = 4)	Dengue virus (*n* = 34)	Measles virus (*n* = 49)	Parvovirus B19 (*n* = 35)	Roseola virus (*n* = 38)	Zika virus (*n* = 3)	Unknown^*b*^ (*n* = 37)	Total (*n* = 200)	
Captia	0 (100)	3 (91.2)	3 (93.9)	1 (97.1)	1 (97.4)	0 (100)	2 (94.6)	10 (95.0)	90.7–97.4
Enzygnost	0 (100)	5 (85.3)	9 (81.6)	4 (88.6)	2 (94.7)	0 (100)	1 (97.3)	21 (89.5^*c*^)	84.2–93.2
Euroimmun	1 (75)	2 (94.1)	6 (87.8)	4 (88.6)	1 (97.4)	0 (100)	3 (91.9)	17 (91.5^*c*^)	86.5–94.8
Euroimmun Glycoprotein	0 (100)	1 (97.1)	4 (91.8)	1 (97.1)	1 (97.4)	0 (100)	1 (97.3)	8 (96.0)	92.0–98.1
Liaison^*d*^	0 (100)	1 (87.5)	0 (100)	0 (100)	2 (94.3)	0 (100)	0 (100)	3 (97.9)	93.4–99.4
Microimmune	0 (100)	1 (97.1)	2 (95.9)	0 (100)	1 (97.4)	0 (100)	0 (100)	4 (98.0)	94.6–99.4
NovaLisa	0 (100)	0 (100)	0 (100)	0 (100)	1 (97.4)	0 (100)	0 (100)	1 (99.5^*c*^)	96.8–100.0
Serion (activity calculator)^*e*^	0 (100)	7 (79.4)	10 (79.6)	17 (51.4)	2 (94.7)	0 (100)	3 (91.9)	39 (80.5^*c*^)	74.2–85.6
Serion (OD range)^*e*^	0 (100)	7 (79.4)	10 (79.6)	17 (51.4)	3 (92.1)	0 (100)	3 (91.9)	40 (80.0^*c*^)	73.6–85.2
Serion (special case formula)^*e*^	0 (100)	7 (79.4)	10 (79.6)	17 (51.4)	2 (94.7)	0 (100)	3 (91.9)	39 (80.5^*c*^)	74.2–85.6

a Specimens with equivocal results were counted as positive.

b This panel included sera from cases with fever/rash illness of unknown etiology.

c Significant difference (*P* < 0.05) between the most specific (NovaLisa) and less specific methods (Enzygnost, Euroimmun, and all three Serion result determination methods) based on nonoverlapping 95% confidence intervals.

d The number of specimens tested with the Liaison method deviated as follows: dengue, *n* = 8; measles, *n* = 20; roseola, *n* = 33; total, *n* = 140.

e Three methods of sample result determination, using the single set of optical density data from the test plates, were provided in the manufacturer’s IFU. All three methods were evaluated.

### Cross-reactivity.

**FIG 1:**
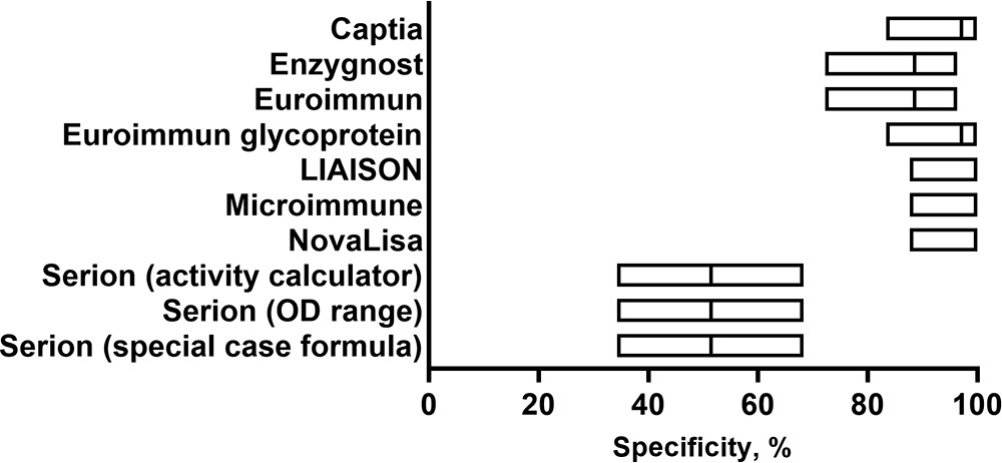
Calculated specificity and 95% confidence intervals for the rubella IgM detection methods evaluated with the parvovirus B19 sera specifically (*n* = 35).

All sera in the non-rubella serum panel either were IgM positive for other agents causing illnesses that can present with fever and rash symptoms (*n* = 163 sera) or were collected from individuals reported as having fever and rash (*n* = 37) (Table 1). To assess possible cross-reactivity with any specific agent, the number of positive or equivocal results by subset was determined (Table 4). Sera collected from cases of roseola or fever/rash illness of unknown etiology had negligible numbers of positive or equivocal results with the methods evaluated. Sera collected from cases of measles demonstrated some potential cross-reactivity, notably with the Euroimmun (6/49), Enzygnost (9/49), and Serion (10/49) methods. The Serion method (all three result determination methods) demonstrated high potential cross-reactivity with sera from dengue (7/34) and measles (10/49) and with half of the parvovirus B19 (17/35) sera evaluated. The specificity of the Serion method, when calculated for the parvovirus B19 sera alone (51.4%; 95% CI, 34.3 to 68.3%), was significantly worse than those of all other methods evaluated (Fig. 1).

### Clinical accuracy.

The classification of the sera as either true rubella positive or negative (84% of the sera were confirmed for an illness with fever and rash of another etiology) (Table 1) was used to determine how accurately each method identified the two classifications of sera (Table 5). When an always-wrong approach was used to classify equivocal test results, the reference standard Enzygnost method had a clinical accuracy of 90.8% (95% CI, 86.2% to 94.0%) and a kappa measure of concordance of 0.716 (95% CI, 0.607 to 0.825). Five methods exceeded the Enzygnost kit: Captia (92.4%), NovaLisa (93.7%), Liaison (94.8%), Euroimmun glycoprotein (95.0%) and Microimmune (95.0%). The Euroimmun glycoprotein and Microimmune methods had statistically significantly better accuracies than that of the Serion kit (all three result determination methods). Only the Microimmune, Euroimmun glycoprotein, and Liaison assays had kappa statistics exceeding 0.8, indicating superior agreement between the test result and the true result. The kappa statistic for the least accurate method, Serion (all three result determination methods), was only 0.55, indicating only moderate agreement.

**TABLE 5:** Calculated clinical accuracy and kappa statistic for concordance of the commercial methods for the detection of anti-rubella IgM antibodies against the predetermined classification of the rubella and non-rubella sera (*n* = 38 and 200, respectively)

Assay	Rubella specimens with equivocal results counted as negative^*a*^				All specimens with equivocal results counted as positive^*b*^			
	Accuracy (%)	95% CI^*c*^	Kappa	95% CI^*c*^	Accuracy (%)	95% CI^*c*^	Kappa	95% CI^*c*^
Captia	92.4	88.1–95.3	0.724	0.604–0.844	93.3	89.1–96.0	0.760	0.648–0.872
Enzygnost	90.8	86.2–94.0	0.716	0.607–0.825	91.2^*d*^	86.6–94.3	0.731	0.626–0.837
Euroimmun	88.7	83.8–92.3	0.607	0.473–0.741	91.2^*d*^	86.6–94.3	0.711^*e*^	0.596–0.826
Euroimmun Glycoprotein	95.0^*d*^	91.1–97.2	0.820	0.721–0.918	96.2	92.7–98.1	0.869	0.785–0.952
Liaison^*f*^	94.8	90.1–97.5	0.830^*g*^	0.722–0.937	96.6	92.3–98.6	0.890	0.804–0.976
Microimmune	95.0^*d*^	91.1–97.2	0.804	0.697–0.911	97.5^*d*^	94.3–99.0	0.908^*e*^	0.836–0.981
NovaLisa	93.7	89.6–96.3	0.727	0.598–0.856	96.2	92.7–98.1	0.848	0.751–0.944
Serion (activity calculator)^*g*^	83.2^*d*^	77.7–87.6	0.554^*g*^	0.440–0.668	83.6^*d*^	78.2–88.0	0.569^*e*^	0.457–0.680
Serion (OD Range)^*g*^	82.8^*d*^	77.2–87.2	0.547^*g*^	0.433–0.660	83.2^*d*^	77.7–88.6	0.561^*e*^	0.449–0.672
Serion (special case formula)^*g*^	83.2^*d*^	77.7–87.6	0.554^*g*^	0.440–0.668	83.6^*d*^	78.2–88.0	0.569^*e*^	0.457–0.680

a Specimens in the rubella serum panel with equivocal results were counted as negative and specimens in the non-rubella serum panel with equivocal results were counted as positive. In this scenario, equivocal results are considered to be always wrong.

b All specimens (rubella and non-rubella) with equivocal results were counted as positive. In this scenario, equivocal results were considered correct for the rubella serum panel and incorrect for the non-rubella serum panel.

c CI, confidence interval.

d Significant difference (*P* < 0.05) between the most accurate and less accurate methods based on nonoverlapping 95% confidence intervals. Using an always-wrong approach for the equivocal results, the Euroimmun Glycoprotein and Microimmune kits were significantly more accurate than the Serion kit (all three result determination methods). With a presumptive positive approach for the equivocal results, the Microimmune kit was most accurate and significantly better than the Enzygnost, Euroimmun, and Serion kits (all three result determination methods).

e Significant difference (*P* < 0.05) from the method with the best kappa statistic for concordance based on nonoverlapping 95% confidence intervals. Using an always-wrong approach for the equivocal results, the Liaison method had a significantly better kappa statistic for concordance than the Serion kit (all three result determination methods). With a presumptive positive approach for the equivocal results, the Microimmune kit had a significantly better kappa statistic for concordance than the Euroimmun and Serion kits (all three result determination methods).

f The total numbers of rubella and nonrubella specimens tested with the Liaison method were 34 and 140, respectively, for a total panel size of 174.

g Three methods of sample result determination, using the single set of optical density data from the test plates, were provided in the manufacturer’s IFU. All three methods were evaluated.

When equivocal results were classified as presumptive positives, the accuracy of all methods improved such that, in addition to the same five methods that exceeded the accuracy of the Enzygnost kit (91.2%), the Euroimmun kit had an equivalent accuracy (Table 5). In this scenario, the Microimmune method was the most accurate (97.5%) and was statistically better than the Enzygnost, Euroimmun, and Serion kits (all three result determination methods). It also had a kappa statistic of 0.908, indicating excellent concordance with the true positive or negative classification, which was statistically better than that of the Euroimmun and Serion methods. The Serion method showed marginal improvement in both accuracy and kappa statistic for concordance when equivocal results were classified as presumptive positives.

### Serum reactivities and receiver-operator characteristic curves for ELISA methods.

**FIG 2:**
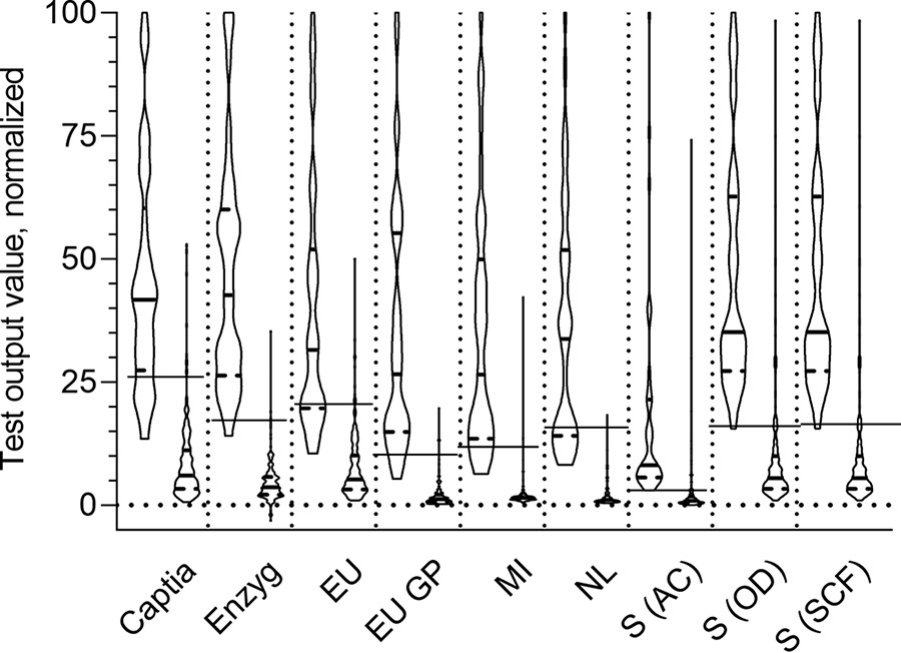
Violin plot of output values for each method by sample set, normalized to 100. Due to different test method OD data manipulations generating output values with different scales, the maximum value for each test kit was set to 100 and all other values were normalized to this value. Each pair corresponds to a test method, with the left plot of each pair representing the output data for the rubella samples and the right plot the non-rubella samples. The horizontal bar across each pair indicates the cutoff for classifying positive results; sera with values above the bar are classified as positive and those below as equivocal (immediately below) or negative. Heavy solid lines within the violin plots are median values, and dashed lines above and below are quartiles. The Liaison method was excluded because a result of <10 was obtained for most of the non-rubella sera. Enzyg, Enzygnost; EU, Euroimmun; EU GP, Euroimmun glycoprotein; MI, Microimmune; NL, NovaLisa; S (AC), Serion activity calculator result determination method; S (OD), Serion OD range result determination method; S (SCF), Serion special case formula result determination method.

All methods generated test results as continuous variables (usually the result of mathematical calculations of the resulting OD value, such as dividing the OD by that of a calibrator control, as specified in the IFU) which were then compared to cutoff values to convert the numerical value to a qualitative result. (The majority of the nonrubella panel specimens resulted in “<10” results with the Liaison method; therefore, they were excluded from this analysis.) To correct for differences of scale across methods, the data were normalized to 100 and plotted for comparison across methods and between panels (rubella and non-rubella) (Fig. 2). Most of the methods demonstrated a clear divide between the test values for the rubella and nonrubella specimens. The Euroimmun glycoprotein, Microimmune, and NovaLisa methods had consistently low values with the non-rubella specimens that were clearly distinguishable from that of the rubella specimens. The positive cutoff value for these methods was generously above the values for most of the non-rubella specimens and, for NovaLisa in particular, resulted in many rubella specimens falling under the cutoff (Fig. 2).

ROC curves were generated and analyzed for each ELISA method (see Fig. S1 and Tables S1 and S2 in the supplemental material). With this data set, a maximum combined sensitivity and specificity could be achieved by reducing the positive cutoff for several methods, as seen also in the violin plots (Table S2; Fig. 2). For the Captia, Euroimmun glycoprotein, and Microimmune methods, better sensitivity and specificity metrics could be obtained by lowering the positive cutoff to the equivocal/negative cutoff. With this data set, dramatically lowering the positive cutoff for the NovaLisa method would result in optimal sensitivity and specificity (both >95%), while using the cutoff specified in the IFU achieved 99.5% specificity at the expense of sensitivity (63.2%). The ROC analysis demonstrated that the Serion method (all three result determination methods), on the other hand, would achieve a better balance of sensitivity and specificity (both ∼92%) with a higher positive cutoff.

### Review of external positive-control results (repeatability and reproducibility).

An external positive control (one for the CLIA method and another for all ELISA methods) was included in every test run. Averages, standard deviations, and coefficients of variation (CV) were calculated for test runs that included at least two replicates of the control (intra-assay) and between runs (interassay) (Table 6). Most methods had excellent intra-assay repeatability, with CV values below 5%. The intra-assay CV deviated widely for both Euroimmun kits (0.8% to 16.3% and 1.5% to 11.5%) and the NovaLisa kit (0.5% to 18.0%). As expected, the interassay reproducibility was lower, with only two methods (Enzygnost and Serion, specifically the OD range and special case formula result determination methods) having a CV less than 5% (4.3%, 2.2%, and 2.2%, respectively). With the exception of the Captia and Euroimmun glycoprotein kits, all methods had a good interassay CV of 10% or under. The Enzygnost, Microimmune, and Serion activity calculator methods had sizeable increases (>5-fold) in their interassay CVs compared to their intra-assay CVs, which was especially unexpected given their extremely low intra-assay CVs. While only one lot of kits was included in the evaluation for most methods, Enzygnost and Microimmune both had two lots evaluated, perhaps accounting for the unexpectedly higher interassay CV.

**TABLE 6:** Repeatability and reproducibility of the commercial methods for the detection of anti-rubella virus IgM antibodies, evaluated by intra- and interassay coefficients of variation of an external positive control included on all test plates^*a*^

Method	Intraassay coefficient of variation (%) (*n*)								Interassay coefficient of variation (%) (*n*)
	1	2	3	4	5	6	7	8	
Captia	NA (1)	NA (1)	NA (1)	NA (1)	ND	ND	ND	ND	11.1 (4)
Enzygnost	0.1 (2)	1.7 (2)	1.9 (2)	0.8 (2)	0.1 (2)	0.5 (2)	ND	ND	4.3 (6)
Euroimmun	0.8 (2)	6.7 (2)	16.3 (2)	ND	ND	ND	ND	ND	8.4 (3)
Euroimmun glycoprotein	1.5 (2)	11.5 (2)	7.9 (2)	ND	ND	ND	ND	ND	12.4 (3)
Liaison	2.9 (3)	4.3 (3)	4.6 (3)	3.7 (3)	ND	ND	ND	ND	6.4 (4)
Microimmune	2.2 (2)	1.9 (2)	1.2 (2)	2.5 (2)	0.1 (2)	0.3 (2)	NA (1)	NA (1)	9.7 (8)
NovaLisa	18.0 (2)	0.5 (2)	5.3 (2)	ND	ND	ND	ND	ND	8.1 (3)
Serion (activity calculator)	1.4 (2)	2.2 (2)	0.4 (2)	ND	ND	ND	ND	ND	8.5 (3)
Serion (OD range)	0.9 (2)	1.4 (2)	0.2 (2)	ND	ND	ND	ND	ND	2.2 (3)
Serion (special case formula)^*b*^	0.9 (2)	1.4 (2)	0.2 (2)	ND	ND	ND	ND	ND	2.2 (3)

a The same external positive control was used for all methods, with the exception of the Liaison method, which used a second control. NA, not applicable; ND, not determined.

b Three methods of sample result determination, using the single set of optical density data from the test plates, were provided in the manufacturer’s IFU. All three methods were evaluated.

## DISCUSSION

Detection of rubella IgM antibodies has been a long-standing and widely used method for rubella laboratory confirmation and surveillance. Previous evaluations have led to the broad adoption of the Enzygnost ELISA method within the WHO global measles and rubella laboratory network (8, 9). With the discontinuation of these kits, suitable alternatives need to be identified. This study evaluated six comparable ELISA methods from five different manufacturers and one automated CLIA method (Liaison). None of the ELISA methods evaluated demonstrated both high sensitivity and specificity with the panel of samples available for this study. Many had high sensitivity (Serion) or high specificity (NovaLisa, Microimmune, and Captia) but not both. The reference method, Enzygnost, and the Euroimmun glycoprotein method were the two methods where the trade-off between the two performance characteristics was more acceptable (sensitivity and specificity of 97.4 and 89.5% and of 89.5 and 96.0%, respectively). The situation improved when specimens with an equivocal result were considered to be presumptively positive, in recognition of the fact that surveillance programs in rubella elimination settings operate with high sensitivity. Cases with equivocal results would often be reflexed for additional workup, such as IgG avidity. In this case, the Euroimmun glycoprotein and Microimmune ELISA methods had excellent sensitivity (97.4% and 94.7%, respectively) and specificity (96.0% and 98.0%, respectively). This was also reflected in their clinical accuracies (96.2% and 97.5%, respectively) and kappa measures of concordance (0.869 and 0.908, respectively).

It is possible that the performance characteristics of some of the methods evaluated in this study could be improved by an adjustment of the selection of the cutoff used to define a positive result. For some methods, the ROC curve data suggested that performance could be improved by lowering the positive cutoff to that of the equivocal range, or even more (NovaLisa). However, this was one small study, using archival specimens, and further evaluations should be performed, using much larger sample sets with well-characterized specimens, before such adjustments should be considered.

IgM capture methods are often seen as being more specific than indirect antibody detection methods. This study evaluated three capture methods (Liaison, Microimmune, and NovaLisa), while the remaining methods were indirect ELISAs. These methods did indeed have the highest specificity of all methods evaluated; however, their sensitivities were mediocre. For the Liaison and Microimmune methods, the poor sensitivity could be compensated for by applying a presumptively positive approach to specimens with equivocal results.

There are several possible reasons for poor specificity for serological tests, one of which is cross-reactivity with antibodies that are directed to other antigens (20). This study included in the non-rubella panel sera collected from confirmed cases of infection with other viral agents causing illnesses that may present with symptoms similar to those of rubella (fever and rash). Sera from these cases may be subjected to rubella IgM testing as part of the differential diagnosis strategy or captured through case finding algorithms (7, 8, 11–15). The Serion ELISA method in particular was prone to cross-reactivity with sera from cases of dengue, measles and parvovirus B19 infection. The panel of measles sera included in this evaluation (*n* = 49) resulted in a significant number of positive or equivocal results with the Euroimmun, Enzygnost, and Serion methods. This is of particular concern because the WHO global measles and rubella laboratory network advocates routine testing of suspected measles or rubella cases for both measles and rubella IgM (8). The identification of double positives (measles and rubella) leads to uncertainty regarding the classification of cases within surveillance systems.

This study had some limitations, including the small size of the panels, particularly for the rubella sera. Rubella has been eliminated in Canada and in many other countries for many years (1, 2), and thus, acquiring sera from confirmed acute cases of rubella is challenging. As a result, this study relied on archival sera, many of which date to the 1990s, and sera sourced commercially, marketed as external positive controls. However, to control for the age and storage history of the sera, sera without a positive result in the majority of methods were excluded from analysis. Using this criterion, only one specimen was excluded. The difficulty in sourcing acute-phase sera from confirmed rubella cases, as well as sera from confirmed acute cases of measles, also contributed to the volume that was available for use in this study. In addition, it reduced the number of specimens that could be used to evaluate the automated Liaison method, which required a significant dead volume and prevented the ability to evaluate the reproducibility of the results through repetition of the panel on additional lots of the evaluated assays.

In summary, none of the methods for detecting anti-rubella IgM antibody evaluated in this study had the combination of high sensitivity and high specificity that is needed to support monitoring of elimination goals, although this could be somewhat compensated for by taking a presumptively positive approach to specimens with equivocal results (particularly Microimmune and Euroimmun glycoprotein methods). These underperforming methods, when combined with low rubella prevalence in elimination settings, lead to poor predictive ability and are therefore of limited value (5, 6, 21–23). A study conducted in the province of Ontario, Canada, a setting without endemic circulation of rubella, reported that the positive predictive value of a positive rubella IgM test for case confirmation was 3.6% (22). Furthermore, in the event of rubella IgM testing when not clinically indicated, such as in screening of pregnant women, the test results arguably do more harm than good. One recent study from the national reference laboratory in France estimated the positive predictive value of rubella IgM serology used to assess maternal primary rubella infection at only 1.4% (23). In these settings, alternative laboratory diagnostic methods with higher specificity and sensitivity, such as viral detection by RT-PCR, which has the added benefit of facilitating viral genotypic surveillance, are preferable for case confirmation.
