# Supplementary material for: Evaluation of Diagnostic Accuracy of Eight Commercial Assays for the Detection of Rubella Virus-Specific IgM Antibodies
Source: J Clin Microbiol. 2022 Jan 19;60(1):e01597-21. doi: 10.1128/JCM.01597-21 (PMC8769748; doi:10.1128/JCM.01597-21)
Supplement: Supplemental file 1 — Fig. S1 and Tables S1 and S2. Download JCM.01597-21-s0001.pdf, PDF file, 0.9 MB [file jcm.01597-21-s0001.pdf]

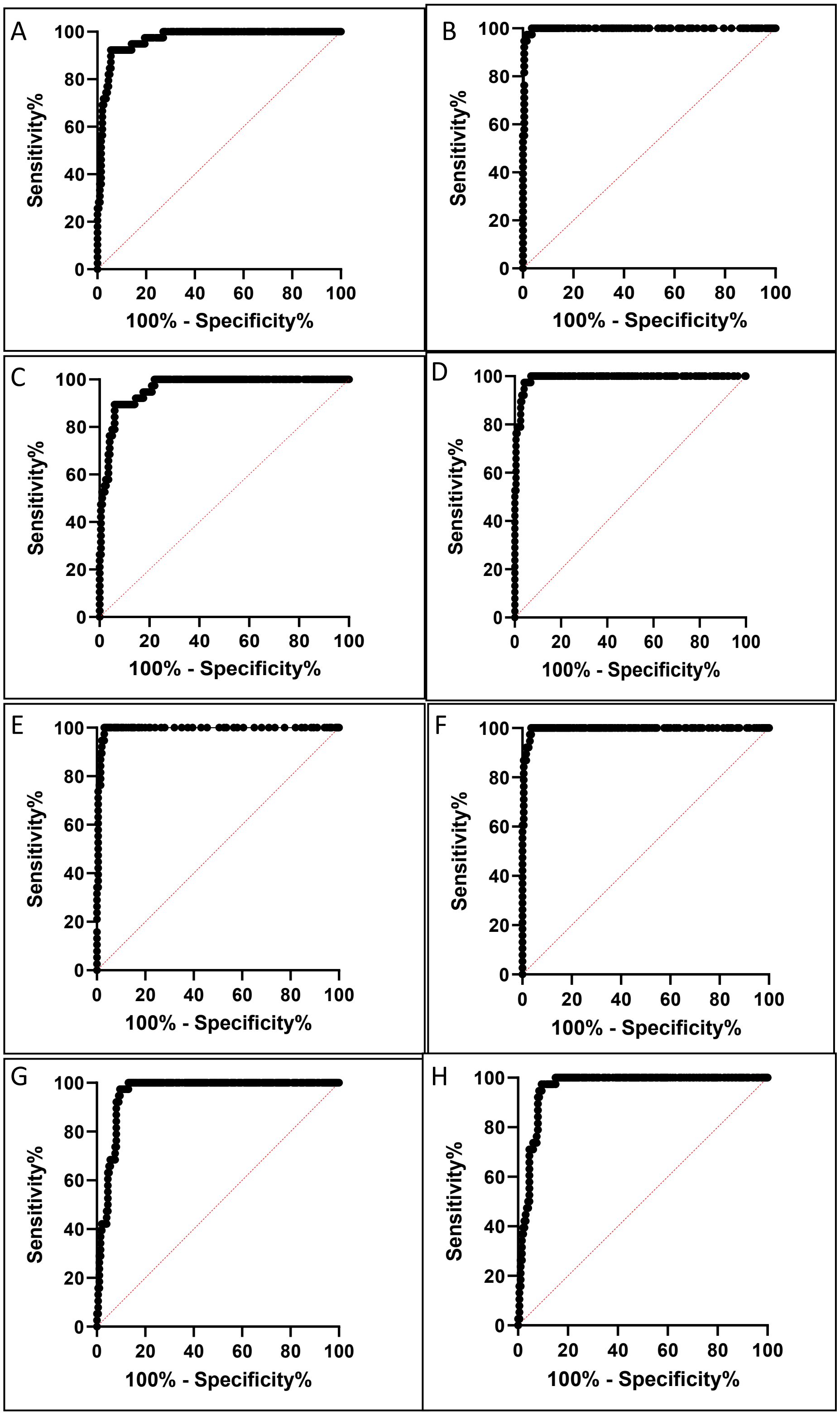

Supplementary Figure 1. ROC curves for the data from the 7 rubella IgM ELISA methods evaluated with the panel of 238 sera. Methods from left to right, top to bottom are: Captia (A), Enzygnost (B), Euroimmun (C), Euroimmun glycoprotein (D), Microimmune (E), NovaLisa (F) and Serion (G & H). For Serion (G & H), three methods of result determination were included in the IFU (activity calculator, OD range and special case formula) and all three were evaluated. The ROC curve for the activity calculator method is at the bottom left (G). The bottom right is the ROC curve for both the OD range method and special case formula as they used identical data (H).

Supplementary Table 1. ROC curve statistics for the 7 rubella IgM ELISA methods evaluated with the panel of 238 sera.

	**Captia**	**Enzygnost**	**Euroimmun**	**Euroimmun GP**	**Microimmune**	**NovaLisa**	**Serion (Activity Calculator)[a]**	**Serion (OD Range)[a]**	**Serion (Special Case Formula)[a]**
Area under the ROC curve									
Area	0.9738	0.9967	0.9631	0.9912	0.9932	0.9955	0.9569	0.9586	0.9586
Std. Error	0.009354	0.002535	0.01175	0.004198	0.004019	0.002736	0.01205	0.01181	0.01181
95% confidence interval	0.9555 to 0.9922	0.9917 to 1.000	0.9401 to 0.9861	0.9830 to 0.9994	0.9853 to 1.000	0.9901 to 1.000	0.9333 to 0.9805	0.9354 to 0.9817	0.9354 to 0.9817
P value	<0.0001	<0.0001	<0.0001	<0.0001	<0.0001	<0.0001	<0.0001	<0.0001	<0.0001
Data									
Controls (Not rubella)	200	200	200	200	200	200	199	200	200
Patients (Rubella)	38	38	38	38	38	38	38	38	38
Missing Controls	0	0	0	0	0	0	0	0	0
Missing Patients	0	0	0	0	0	0	0	0	0

[a] Three methods of sample result determination, using the single set of optical density data from the test plates, were provided in the manufacturer's IFU. All three methods were evaluated.

Supplementary Table 2. Calculated sensitivity and specificity values for select cut-offs from the ROC curve analysis of the data from the 7 rubella IgM ELISA methods evaluated with the panel of 238 sera.

Method	Description	Positive Cut-off	Sensitivity (%)	95% CI	Specificity (%)	95% CI	Likelihood ratio
Captia	IFU positive	≥ 1.10	78.95	63.65% to 88.93%	96	92.31% to 97.96%	19.74
	IFU equivocal	> 0.90	86.84	72.67% to 94.25%	95	91.04% to 97.26%	17.37
	Sensitivity ≥95%	> 0.6328	97.37	86.51% to 99.87%	86	80.51% to 90.13%	6.955
	Specificity ≥95%	> 0.8935	86.84	72.67% to 94.25%	95	91.04% to 97.26%	17.37
	Max. sens. & spec.[b]	> 0.8464	94.74	82.71% to 99.06%	94.5	90.42% to 96.90%	17.22
Enzygnost	IFU positive	> 0.2	94.74	82.71% to 99.06%	98.5	95.68% to 99.59%	63.16
	IFU equivocal	≥ 0.1	100	90.82% to 100.0%	89.5	84.48% to 93.03%	9.524
	Sensitivity ≥95%	> 0.1970	97.37	86.51% to 99.87%	98.5	95.68% to 99.59%	64.91
	Specificity ≥95%	> 0.1305	100	90.82% to 100.0%	95.5	91.67% to 97.61%	22.22
	Max. sens. & spec. [b]	> 0.1970	97.37	86.51% to 99.87%	98.5	95.68% to 99.59%	64.91
Euroimmun	IFU positive	≥1.1	73.68	57.99% to 85.03%	96	92.31% to 97.96%	18.42
	IFU equivocal	≥0.8	89.47	75.87% to 95.83%	91.5	86.81% to 94.63%	10.53
	Sensitivity ≥95%	> 0.5965	97.37	86.51% to 99.87%	79	72.84% to 84.07%	4.637
	Specificity ≥95%	> 0.9889	78.95	63.65% to 88.93%	95	91.04% to 97.26%	15.79
	Max. sens. & spec. [b]	> 0.8852	89.47	75.87% to 95.83%	94	89.81% to 96.53%	14.91
Euroimmun GP	IFU positive	≥1.1	89.47	75.87% to 95.83%	97.5	94.28% to 98.93%	35.79
	IFU equivocal	≥0.8	97.37	86.51% to 99.87%	96	92.31% to 97.96%	24.34
	Sensitivity ≥95%	> 0.7955	97.37	86.51% to 99.87%	96	92.31% to 97.96%	24.34
	Specificity ≥95%	> 0.7275	97.37	86.51% to 99.87%	95	91.04% to 97.26%	19.47
	Max. sens. & spec. [b]	> 0.7955	97.37	86.51% to 99.87%	96	92.31% to 97.96%	24.34
Microimmune	IFU positive	≥0.35	78.95	63.65% to 88.93%	98.5	95.68% to 99.59%	52.63
	IFU equivocal	≥0.2	94.74	82.71% to 99.06%	98	94.97% to 99.22%	47.37
	Sensitivity ≥95%	> 0.1805	97.37	86.51% to 99.87%	97	93.61% to 98.62%	32.46
	Specificity ≥95%	> 0.09800	100	90.82% to 100.0%	95	91.04% to 97.26%	20
	Max. sens. & spec. [b]	> 0.1630	100	90.82% to 100.0%	97	93.61% to 98.62%	33.33
NovaLisa	IFU positive	> 11	63.16	47.28% to 76.62%	99.5	97.22% to 99.97%	126.3
	IFU equivocal	≥ 9	78.95	63.65% to 88.93%	99.5	97.22% to 99.97%	157.9
	Sensitivity ≥95%	> 5.739	97.37	86.51% to 99.87%	97	93.61% to 98.62%	32.46
	Specificity ≥95%	> 5.321	100	90.82% to 100.0%	95	91.04% to 97.26%	20
	Max. sens. & spec. [b]	> 5.739	97.37	86.51% to 99.87%	97	93.61% to 98.62%	32.46
Serion (Activity Calculator) [a]	IFU positive	> 3.5	97.37	86.51% to 99.87%	88.44	83.25% to 92.17%	8.424
	IFU equivocal	> 2.5	100	90.82% to 100.0%	80.9	74.88% to 85.76%	5.237
	Sensitivity ≥95%	> 4.093	97.37	86.51% to 99.87%	90.45	85.57% to 93.80%	10.2
	Specificity ≥95%	> 7.537	63.16	47.28% to 76.62%	95.48	91.63% to 97.60%	13.96
	Max. sens. & spec. [b]	> 4.093	97.37	86.51% to 99.87%	90.45	85.57% to 93.80%	10.2

Serion (OD Range) [a]	IFU positive	Variable; as tested > 0.39 - 0.45	97.37 - 100		85 - 90.5		
	IFU equivocal	Variable; as tested ≥ 0.29 - 0.34	100		78.5 - 83		
	Sensitivity ≥95%	> 0.4735	97.37	86.51% to 99.87%	90.5	85.64% to 93.83%	10.25
	Specificity ≥95%	> 0.7630	71.05	55.24% to 83.00%	95	91.04% to 97.26%	14.21
	Max. sens. & spec. [b]	> 0.5910	92.11	79.20% to 97.28%	92	87.40% to 95.02%	11.51
Serion (Special Case Formula) [a]	IFU positive	Variable; as tested > 0.397 - 0.459	97.37		85 - 90.5		
	IFU equivocal	Variable; as tested ≥ 0.296 - 0.343	100		80 - 83		
	Sensitivity ≥95%	> 0.4735	97.37	86.51% to 99.87%	90.5	85.64% to 93.83%	10.25
	Specificity ≥95%	> 0.7630	71.05	55.24% to 83.00%	95	91.04% to 97.26%	14.21
	Max. sens. & spec. [b]	> 0.5910	92.11	79.20% to 97.28%	92	87.40% to 95.02%	11.51

[a] Three methods of sample result determination, using the single set of optical density data from the test plates, were provided in the manufacturer's IFU. All three methods were evaluated.
[b] Maximum combined sensitivity and specificity
